# Filling the gaps: Monitoring Casparian strip integrity in rice

**DOI:** 10.1093/plcell/koad274

**Published:** 2023-11-01

**Authors:** Nicolas M Doll

**Affiliations:** Assistant Features Editor, The Plant Cell, American Society of Plant Biologists; Laboratoire Reproduction et Développement des Plantes, University of Lyon, ENS de Lyon, UCB Lyon 1, CNRS, INRAE, F-69342, Lyon, France

Selective uptake of mineral ions from the soil is crucial for plants to control electrolyte levels. In vascular plant roots, the diffusion of water and mineral nutrients through the apoplast is impeded by the Casparian strip, a band of polymerized lignin that seals the apoplastic space around the endodermis. A continuous Casparian strip is critical for the plant to control the passage of water and ions from the cortex into the stele and what is subsequently transported through the vasculature toward the shoot (Geldner 2013).

In Arabidopsis, a specific signaling pathway monitors the integrity of the Casparian strip. Small sulfated peptides, which are encoded by the *CASPARIAN STRIP INTEGRITY FACTOR 1* and *2* (*AtCIF1/2*) genes, are specifically secreted from the root stele (Fig. 1A). If the Casparian strip is discontinuous, these peptides diffuse toward the endodermis, where they can bind to the leucine-rich repeat receptor-like kinase SCHENGEN3 (AtSNG3). This interaction allows the recruitment of the co-receptor SCHENGEN1 (AtSGN1), specifically located at the cortical side of the endodermis. The downstream signaling cascade activates the production of lignin and clogging of the gaps (Fig. 1A). When the Casparian strip is continuous, the AtSGN3/AtSGN1 receptors become physically separated from the AtCIF peptides, and the signaling stops (Doblas et al. 2017; Nakayama et al. 2017).

**Figure 1.**
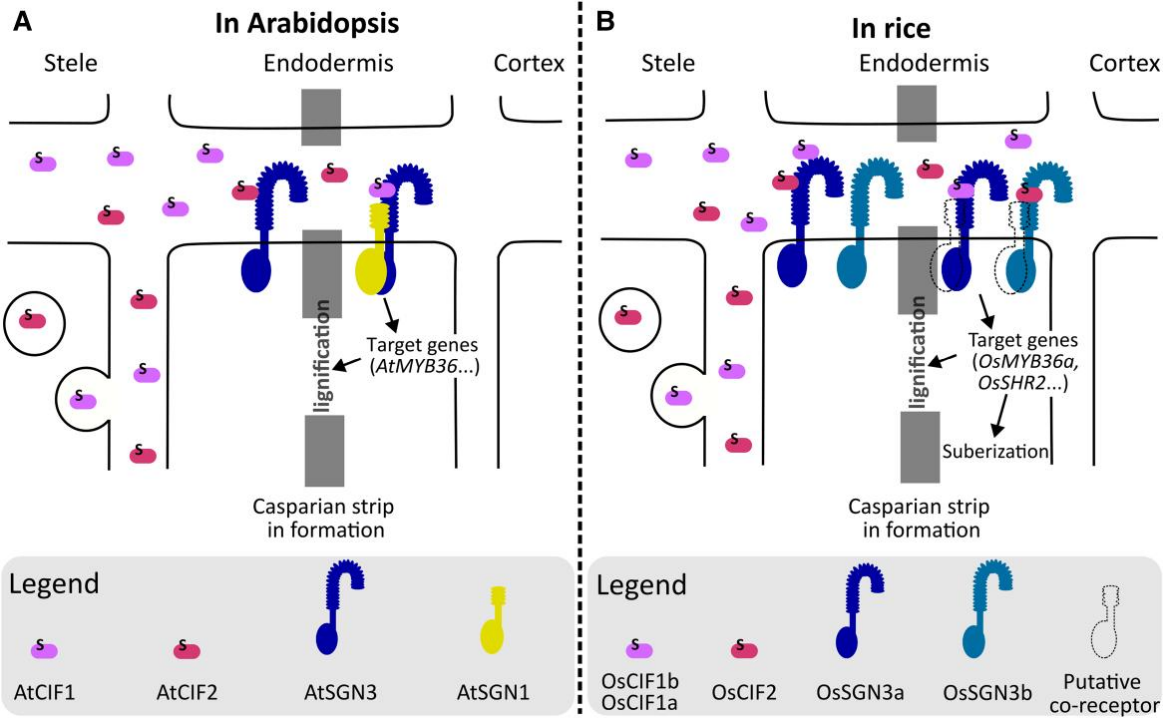
Casparian strip integrity surveillance machinery in Arabidopsis (**A**) and rice (**B**). Sulfated CIF peptides are secreted by the stele, diffuse through discontinuous Casprian strip, and bind the SGN3 receptor(s) in the endodermis. Co-receptor recruitment at the cortical side leads to a signaling cascade that activates target genes involved in Casparian strip lignification and clogging, as well as endodermis suberization specifically in rice. CIF/SGN signaling stops when a continuous Casparian strip blocks the diffusion of the CIF peptides. Figure made by NM Doll with Inkscape.

The CIF/SGN3 signaling pathway thus acts as surveillance machinery for Casparian strip integrity in Arabidopsis. The extent to which this machinery is conserved in other plant species has remained unknown. In this article, **Baolei Zhang and colleagues** (Zhang et al. 2023) investigated the conservation of the CIF/SGN3 pathway in rice. They identified 2 orthologous genes of *AtSGN3*, *OsSGN3a* and *OsSGN3b*, both expressed in the endodermis, and 3 orthologs of the *AtCIF*s, expressed in the stele. *OsCIF1a* and *OsCIF1b* are 2 identical genes expressed in tandem, the third being *OsCIF2*. Loss of function of the 3 CIF peptides in *Oscif1ab,2* triple mutants or the 2 SGN3 receptors in *Ossgn3ab* double mutants resulted in a notably thinner and discontinuous Casparian strip, rendering it more permeable to small apoplastic molecules. Isothermal titration calorimetry experiments showed that both OsCIF1 and OsCIF2 peptides have a strong affinity for the ectodomain of OsSNG3a with dissociation constants of 1600 nM and 439 nm, respectively. Furthermore, RNA-seq analyses conducted in *Oscif1ab,2* and in a line misexpressing *OsCIF1* in the cortex revealed that many orthologs of Arabidopsis genes involved in the Casparian strip formation are expressed downstream of the CIF/SGN3 pathway in rice, such as *OsMYB36a*. Altogether these results indicate that the CIF/SGN3 machinery monitoring Casparian strip integrity is extensively conserved in rice (Fig. 1B).

Interestingly, the authors identified some differences between Arabidopsis and rice. Firstly, in a normal context, the Arabidopsis CIF/SGN3 pathway does not affect the endodermal suberization that follows Casparian strip lignification. In contrast, both *Oscif1ab,2* and *Ossgn3ab* mutants exhibit a clear reduction in endodermal suberization. Secondly, in rice, the misexpression of *OsCIFs* in the cortex induces ectopic lignification and suberization, whereas in Arabidopsis, this only occurs when the transcription factor SHORT ROOT (AtSHR) is misexpressed in the cortex as well. This difference may come from the expression of a second *SHR* (*OsSHR2*) gene in the rice cortex or from OsCIF-induced expression of *OsSHR1* or *OsSHR2*. Thirdly, the CIFs-SHR1 combination can induce the formation of Casparian strip-like structures in more cortical layers in rice than in Arabidopsis, indicating a distinct ability of the CIFs to induce ectopic Casparian strip formation in these two species.

In conclusion, the SGN/CIF pathway plays a crucial role in the formation of a functional Casparian strip in rice and any defects in this pathway have a significant impact on the plant, affecting agronomically important traits such as tiller number, plant height, and grain set. Notably, the shoots of Casparian strip–defective plants display significant alterations in the concentration of various ions, indicating impaired ion homeostasis. This is likely a result of the defective control of ion movement toward the vasculature by a nonfunctional Casparian strip. Interestingly, the aerial parts of the *cif1ab,2* mutant plants are less affected than those of *sgn3ab*, which exhibits male sterility. This indicates additional functions of the OsSGN3 receptors in other contexts, in line with the pleiotropic roles of AtSGN3, such as monitoring embryonic cuticle and pollen coat integrity (Truskina et al. 2022). Further analyses of these functions would provide valuable insights into the conservation of the CIF/SGN3 pathway for the formation of apoplastic barriers between other tissues in rice.

## References

[koad274-B1] Doblas VG , Smakowska-Luzan E, Fujita S, Alassimone J, Barberon M, Madalinski M, Belkhadir Y, Geldner N. Root diffusion barrier control by a vasculature-derived peptide binding to the SGN3 receptor. Science. 2017:355(6322):280–284. 10.1126/science.aaj1562 28104888

[koad274-B2] Geldner N . The endodermis. Annu Rev Plant Biol. 2013:64(1):531–558. 10.1146/annurev-arplant-050312-120050 23451777

[koad274-B3] Nakayama T , Shinohara H, Tanaka M, Baba K, Ogawa-Ohnishi M, Matsubayashi Y. A peptide hormone required for Casparian strip diffusion barrier formation in Arabidopsis roots. Science. 2017:355(6322):284–286. 10.1126/science.aai9057 28104889

[koad274-B4] Truskina J , Brück S, Stintzi A, Boeuf S, Doll NM, Fujita S, Geldner N, Schaller A, Ingram GC. A peptide-mediated, multilateral molecular dialogue for the coordination of pollen wall formation. Proc Natl Acad Sci U S A. 2022:119(22):e2201446119. 10.1073/pnas.2201446119 PMC9295805 35609199

[koad274-B5] Zhang B , Xin B, Sun X, Chao D, Zheng H, Peng L, Chen X, Zhang L, Yu J, Ma D, et al Small peptide signaling via OsCIF1/2 mediates Casparian strip formation at the root endodermal and nonendodermal cell layers in rice. Plant Cell. 2024:36 (2):383–403. 10.1093/plcell/koad269 PMC10827571 37847118

